# Recent advances in virulence of a broad host range plant pathogen *Sclerotinia sclerotiorum*: a mini-review

**DOI:** 10.3389/fmicb.2024.1424130

**Published:** 2024-06-19

**Authors:** Yangyi Zhu, Chenghong Wu, Yun Deng, Wanlan Yuan, Tao Zhang, Junxing Lu

**Affiliations:** Chongqing Key Laboratory of Plant Environmental Adaptations, College of Life Science, Chongqing Normal University, Chongqing, China

**Keywords:** virulence, life cycle, oxalic acid, cell wall degrading enzymes, effector proteins, host-induced gene silencing

## Abstract

*Sclerotinia sclerotiorum* is a typical necrotrophic plant pathogenic fungus, which has a wide host range and can cause a variety of diseases, leading to serious loss of agricultural production around the world. It is difficult to control and completely eliminate the characteristics, chemical control methods is not ideal. Therefore, it is very important to know the pathogenic mechanism of *S. sclerotiorum* for improving host living environment, relieving agricultural pressure and promoting economic development. In this paper, the life cycle of *S. sclerotiorum* is introduced to understand the whole process of *S. sclerotiorum* infection. Through the analysis of the pathogenic mechanism, this paper summarized the reported content, mainly focused on the oxalic acid, cell wall degrading enzyme and effector protein in the process of infection and its mechanism. Besides, recent studies reported virulence-related genes in *S. sclerotiorum* have been summarized in the paper. According to analysis, those genes were related to the growth and development of the hypha and appressorium, the signaling and regulatory factors of *S. sclerotiorum* and so on, to further influence the ability to infect the host critically. The application of host-induced gene silencing (HIGS)is considered as a potential effective tool to control various fungi in crops, which provides an important reference for the study of pathogenesis and green control of *S. sclerotiorum*.

## 1 Introduction

*Sclerotinia sclerotiorum* is a typical necrotrophic nutrient fungus that belongs in taxonomic status to the Fungi, Ascomycota, Discomycetes, Helotiales, Sclerotiniaceae, Sclerotinia. An early study determined that Dictyostelium can infect more than 450 plant species in 75 families, posing a threat primarily to dicotyledonous crops such as sunflowers, soybeans, canola, edible dry beans, chickpeas, peanuts, dry beans, lentils, and a variety of vegetables, but also to monocotyledonous plants such as onions and tulips (Boland and Hall, 1994). Recent research has found that it can grow in rice, wheat, barley, oat, and corn (Tian et al., 2020).

The disease caused by *S. sclerotiorum* is called Sclerotinia. More than 60 names have been used to refer to diseases caused by this fungal pathogen (Purdy, 1979), including cotton rot, water soft rot, stem rot, abscission, crown rot, flower wilt, and the most common white mold. *S. sclerotiorum* is a representative vegetative plant pathogenic fungus with complex pathogenic mechanism. The role and mechanism of oxalic acid, cell wall degrading enzymes, and secretory proteins secreted by *S. sclerotiorum* in the process of infection have been the focus of reports previously. However, the molecular basis of the pathogenesis of *S. sclerotiorum* is imprecise and remains a subject of ongoing research. In this paper, the recent advancements in the virulence of *S. sclerotiorum* have been reviewed, which can serve as an important source of information for molecular research of this organism.

## 2 The infection process of *Sclerotinia Sclerotiorum*

*Sclerotinia sclerotiorum* (Lib.) de Bary is one of the most dangerous and common plant-killing organisms in the world. This fungus may be found all across temperate, tropical, and dry environment (Lehner et al., 2017). Sclerotia consists of three distinct layers, the thick-walled pigmented cortex, the thin-walled cortex and the white medulla (Clarkson et al., 2004). As its name indicates, the fungus produces sclerotia, which are long-lived melanized resting structures. Sclerotia can germinate in two ways, carpogenically to form apothecia from which ascospores are liberated or myceliogenically to produce hyphae. The fungus is homothallic and no asexual spores are produced (Hegedus and Rimmer, 2005). *S. sclerotiorum* generally exists in the form of sclerotia, and the existence of ascospores and mycelium form maintains a shorter time (Bolton et al., 2006). When environmental conditions are fit for germination, sclerotia may germinate myceliogenically to produce hyphae that infect the lower parts of plants, or germinate carpogenically to produce apothecia and release ascospores into the air (Behnam et al., 2007; Fernando et al., 2007; Zhang M. et al., 2021). When infected in the form of ascospores, only combined with multiple infection sites, can the successful infection feature be formed. For example, flowers or flower parts provide an appropriate nutritional basis for the initial colonization of ascospore inoculants. The undamaged host surface is infected by hyphae extending from the matrix (Sutton and Deverall, 1983). Once infection has been established, watery lesions with distinct edges may first appear on stems, leaves, petioles, and reproductive organs. These lesions are then followed by wilting, bleaching, and shredding. Typically on the surface of the infected tissue as well as in soft host tissue or lumen, cotton hyphae build up into pea-sized aggregates that eventually develop into hard, black sclerotia (Heffer and Johnson, 2007). When the stem is completely girdled and covered by the whitish mycelial growth all over, the plant wilts and dries. The fungus produces symptoms on all the aerial plant parts but the most destructive one is produced on the stem. Usually, the plants are attacked at flowering stage and once the pathogen is established in the field, it is very difficult to eliminate it due to the stubborn soil-borne sclerotia and wide host range (Bolton et al., 2006).

Strong pathogenicity enable *S. sclerotiorum* to virtually completely invade all plant tissues with its mycelium. Importantly, the fungus affects hundreds of monocotyledons and dicotyledones and is not restricted to any one host (Jahan et al., 2022). Infection can potentially begin with mycelial development from sclerotia on the soil’s surface. After colonizing dead organic debris, the germinated hyphae infect nearby living plants (Hegedus and Rimmer, 2005). Plant pathogen *S. sclerotiorum* is extremely destructive, and its infection can result in substantial yield loss, the drop in the quantity and quality of affected crops, and other negative economic effects (Fernando et al., 2007).

## 3 Pathogenicity factors

*Sclerotinia sclerotiorum* is a typical necrotrophic plant pathogenic fungus with complex pathogenic mechanism. At present, the main pathogenic factors are classified as plant cell-wall-degrading enzymes(CWDEs), oxalic acid and effector protein secreted by *S. sclerotiorum*. Firstly, *S.sclerotiorum* increases its colonization by secreting CWDEs, which plays an important role in the early stage of infection. Oxalic acid, a key pathogenic factor, is secreted in large quantities in the early stage and plays an important role in pathogenicity. In addition, *S. Sclerotiorum* also secretes some effector proteins to promote infection in its fight against plants.

### 3.1 Oxalic acid, a key factor in the early pathogenesis of *Sclerotinia Sclerotiorum*

**Figure 1:**
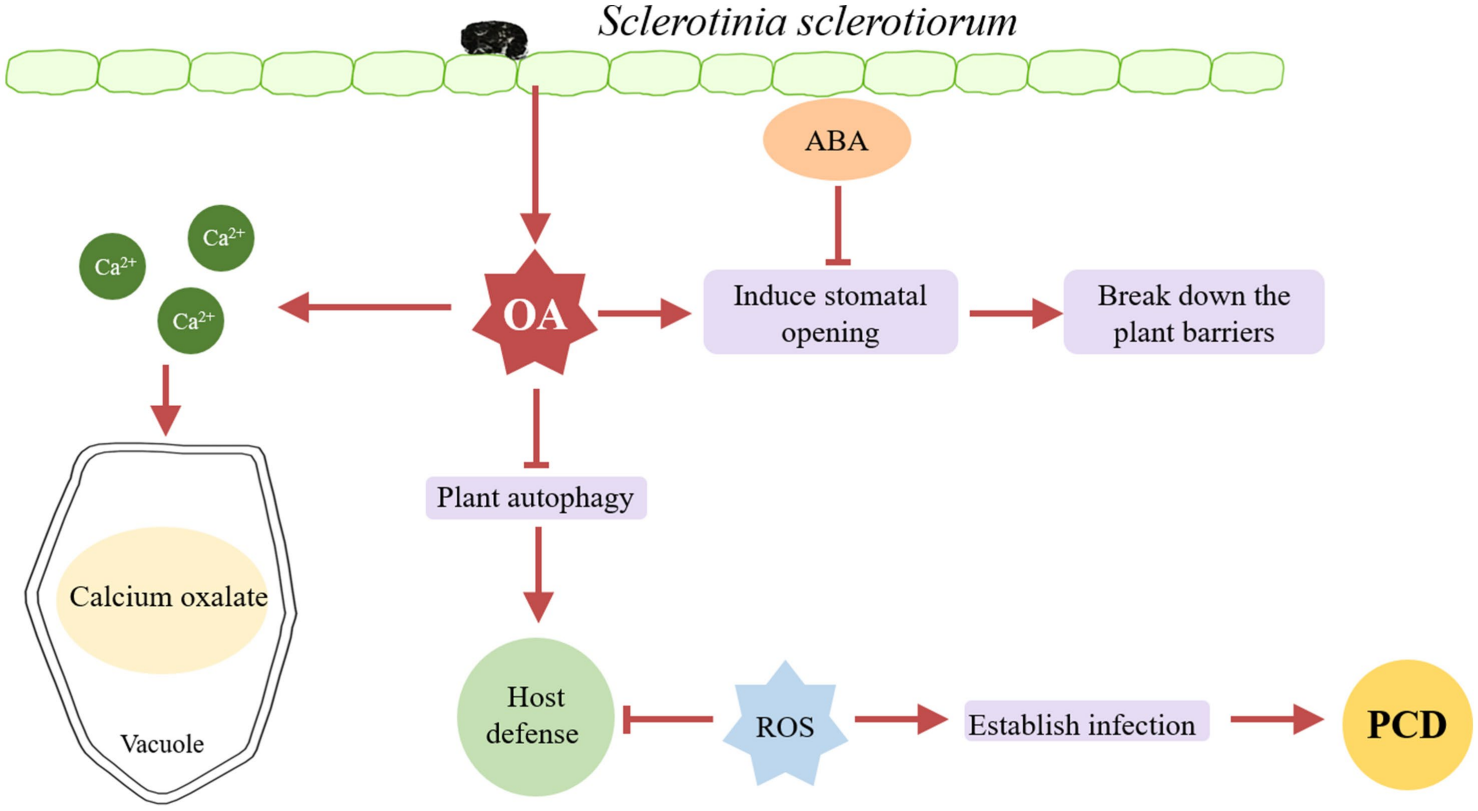
A model of oxalic acid (OA)-induced infection. *S. sclerotiorum* secretes large amounts of oxalic acid during infection. In the early stage of infection, oxalic acid blocks the closure of the plant stomata to promote infection, and then breaks down the plant barrier, at which point the host secretes ABA to shut down the plant stomata to resist infection. Oxalic acid can inhibit plant autophagy, through the outbreak of producing reactive oxygen species (ROS) in the later stage to inhibit the host defense response, and finally cause the programmed cell death (PCD) of plants. In addition, oxalic acid can preserve ca^2+^ generated by cell wall collapsing in the form of calcium oxalate in the vacuole, playing a detoxification role.

Oxalic acid is considered to be a key factor in the early pathogenesis of many necrotrophic fungi. *S. sclerotiorum* involves the production and accumulation of oxalic acid at the early stage of plant susceptibility (Figure 1). Godoy et al. (1990) studied the role of oxalic acid in the pathogenicity of *S. sclerotiorum* in 1990, the oxalic acid-deficient mutants were obtained by UV mutagenesis and lost their pathogenicity. Moreover, the pathogenicity of the mutants was restored after the addition of sodium succinate.

More studies revealed the role of oxalic acid in the pathogenesis of *S. sclerotiorum*. Oxalic acid can produce reactive oxygen species (ROS) and induce programmed cell death (PCD). In contrast, during the initial stage of *S. sclerotiorum* infection of host plants, *S. sclerotiorum* produces a reducing environment in host cells through oxalic acid inhibiting host defense responses, including suppression of ROS bursts and callose deposition, similar to compatible bionutrient pathogens. However, once an infection is established, oxalic acid induces ROS production in the plants, resulting PCD in the host tissue (Williams and Stelfox, 1980). Oxalic acid can regulate the PH of the environment to facilitate the infection of *S. sclerotiorum*. *Sclerotinia sclerotiorum* can cause infection and disease at least in some plants as long as the environmental pH is low enough (Bateman and Beer, 1965). Oxalic acid prevents stomatal closure in the host plant. The results showed that when the leaves of *Vicia faba* were infected with *S. sclerotiorum* mutants lacking oxalic acid, the stomata were partially closed (Guimarães and Stotz, 2004). Besides, Oxalate prevents stomatal closure caused by abscisic acid (ABA). In Arabidopsis, compared to wild type plants, mutants *abi1*, *abi3*, *abi4*, and *aba2* are more vulnerable to oxalate-deficient *S. sclerotiorum*, indicating that ABA is necessary for Sclerotinia resistance (Guimarães and Stotz, 2004). Oxalic acid may be able to quench calcium ions generated during cell wall collapse to shield developing hyphae from the harmful calcium concentrations in the infection region. Heller and Witt-Geiges (2013) suggest that calcium was transferred to the older parts of the mycelium and detoxified by forming nontoxic, stable oxalate crystals and they proposed an infection model in which oxalic acid plays a detoxifying role in the late stage of infection.

### 3.2 Cell wall degrading enzymes play a vital role in pathogenic fungi invading host

Plant cell wall is an important place for interaction between host and pathogenic fungi, and plays a vital role in the process of plant pathogenic fungi invading host. The pathogenic fungi secrete a series of cell wall degrading enzymes during the process of infecting host plants. They can not only take up nutrition, but also degrade host tissue during the pathogenic process of pathogenic fungi. *Sclerotinia sclerotiorum* releases hydrolases, which degrade the cuticle, the middle layer, and the primary and secondary cell walls in turn. It was found that the cuticle-stripped leaves infected more quickly, so the cuticle could act as a barrier against Sclerotinia infection.

*Sclerotinia sclerotiorum* can secrete different cell wall degrading enzymes, especially Polygalacturonases (PGs), when it infects host plants. According to reports, four endo-polygalacturonase (PG) genes (*SsPG1*, *SsPG3*, *SsPG5* and *SsPG6*) and two exo-PGs genes (*SsXPG1* and *SsXPG2*) were identified in *B. napus* during *S. Sclerotiorum* infection (Li et al., 2004). The factors affecting the regulation of genes encoding polygalacturonase 1(*SsPG1*) and a newly identified keratinase (*SsCUTA*) were studied. *In vitro*, *SsCUTA* transcripts were detected within 1 h after leaf inoculation, and their expression was mainly controlled by the contact of mycelium with solid surface. The expression level of the keratinase-encoding gene *SsCUTA* of *S. sclerotiorum* was significantly up-regulated at 1 h after infection. Expression of *SsPG1* was moderately induced by contact with solid surfaces, including leaves, and as infection progresses, expression of *SsPG1* was limited to the extended margins of the lesion (Dallal Bashi et al., 2012). Both *SsPG3* and *SsPG6* induced light-dependent necrosis in *Arabidopsis thaliana* leaves (Dallal Bashi et al., 2012).

### 3.3 Effector proteins, critical components in the effective pathogenesis of *Sclerotinia Sclerotiorum*

In the process of antagonism between plant and pathogen, the pathogen will secrete some proteins into plant cells. These proteins are called “Effector proteins,” which can make the pathogen successfully infect the plant. These “Effector proteins,” have been found in a variety of plant pathogenic fungi and exhibit many different functions according to the life style of the fungi.

*SsITL* encodes a protein containing 302 amino acid residues, which belongs to the integrin-terminal domain superfamily. The expression of *SsITL* gene increased sharply during the early stage of infection and after the gene silencing, the pathogenicity of *S. sclerotiorum* decreased. The host plants overexpressing *SsITL* gene were more susceptible. Further studies showed that *SsITL* protein was involved in the inhibition of JA/ET signaling pathway-mediated local and systemic disease resistance in *S. sclerotiorum*. Therefore, *SsITL* played a similar role as an effector in *S. Sclerotiorum* pathogenesis (Wang et al., 2009). Two genes, *SsNEP1* and *SsNEP2*, encoding necrotic and ethylene-induced polypeptides (NEPs), were identified in *S. sclerotiorum*. During infection, the expression of *SsNEP1* was increased. The expression of *SsNEP2* was induced by contact with solid surfaces and occurs in necrotic areas and the leading edge of infection. Expression of *SsNEP2* was dependent on calcium and cyclic AMP signaling, instead, compounds that affected these pathways reduced or eliminated *SsNEP1* expression with partial or complete loss of virulence (Dallal Bashi et al., 2010). A small cysteine-rich protein, SsCVNH, has been shown to be crucial for virulence and sclerotium development (Lyu et al., 2015a). Another small cysteine-rich protein SsSSVP1 in *S. sclerotiorum* has been identified as a secreted protein and its targeted silencing resulted in reduced virulence (Lyu et al., 2016).

## 4 Reported genes regulating virulence in *Sclerotinia Sclerotiorum*

The virulence of *S. Sclerotiorum* is related to the growth and development of its appressorium and its ability to infect plants. Studies on the virulence of *S. sclerotiorum* to date have been shown as follows (Table 1). In the regulation of pathogenicity in *S. sclerotiorum*, several genes play pivotal roles. *SsTrx1* contributes to enhancing the pathogenicity of the fungus and its tolerance to oxidative stress (Rana et al., 2021), while overexpression of *SsYCP1* promotes *S. sclerotiorum* infection and enhances its pathogenicity (Fan et al., 2021a). *SsNACα* regulates the expression of polygalacturonase, thus impacting the pathogenicity of *S. sclerotiorum* (Li et al., 2015). Additionally, *SsERP1* modulates the virulence of *S. sclerotiorum* by regulating the ethylene pathway (Fan et al., 2021b). Moreover, *SsSte12* influences fungal hyphal growth, adhesion structure formation, and virulence regulation (Xu et al., 2018). *SsPDE2* plays a regulatory role in oxalate accumulation, adhesion structure formation, and virulence (Xu et al., 2023). Furthermore, *SsXyl1*, encoding endo-β-1,4-xylanase, plays a crucial role in sclerotium formation and infection processes (Yu et al., 2016). *SsNsd1* regulates the morphology of *S. sclerotiorum* hyphae, appressorium structures, sclerotium, and pathogenicity (Li et al., 2018).

**Table 1:** Virulence-related genes of *S. sclerotiorum.*

Gene name	Mutant type	Mutant virulence	Tested host	Gene function	Reference
*pac1* (AY005467)	knockout, site-directed mutagenesis	Reduced	*A*.*thaliana*, tomato	Zinc finger transcription factor	Rollins (2003) and Kim et al. (2007)
*Sssac1* (SS1G_07715)	Knockout	Reduced	Tomato	Adenylate cyclase	Jurick and Rollins (2007)
*rgb1*	RNA-silencing	Abolished	*A.thaliana*, tomato	Type 2A phosphoprotein phosphatase	Erental et al. (2007)
*Ssaxp* (SS1G_02462)	Knockout	Reduced	Rapeseed	Arabinofuranosidase/β-xylosidase precursor	Yajima et al., 2009
*Ss-ggt1* (SS1G_14127)	Knockout	Reduced	Tomato	γ-glutamyl transpeptidase	Li et al. (2012)
*Ss-pth2* (SSIG_13339)	Knockout	Reduced	Soybean	Peroxysomal carnitine acetyl transferase	Liberti et al. (2013)
*SsSOD1* (SS1G_00699)	T-DNA insertional	Reduced	Pea	Cu/Zn superoxide dismutase	Xu and Chen (2013)
*SsMADS* (FJ869956)	RNA-silencing	Reduced	Tomato	MADS-box transcription factor	Qu et al. (2014)
*Ss-caf1* (SS1G_02486)	T-DNA insertional	Reduced	*A*.*thaliana*, rapeseed	Encoding a secretory protein with a putative Ca^2+^ binding EF-hand motif	Xiao et al. (2014)
*Ssoah1* (SS1G_08218)	Knockout	Reduced	*A*.*thaliana*, soybean, tomato	oxaloacetate acetylhydrolase gene, key enzyme of oxalic acid	Liang et al. (2015a)
*Ss-odc2* (SS1G_10796)	Knockout	Reduced	Soybean	Oxalate decarboxylase	Liang et al. (2015b)
*SsNACα* (SS1G_05284)	RNA-silencing	Increased	*N.benthamiana*, rapeseed	The nascent polypeptide-associated complex α subunit gene	Li et al. (2015)
*SsXyl1* (SS1G_07749)	Knockout	Reduced	*A*.*thaliana*, rapeseed	Endo-β-1, 4-xylanase	Yu et al. (2016)
*sop1* (SS1G_01614)	RNA-silencing	Reduced	Rapeseed	Microbial opsin homolog gene	Lyu et al. (2015b)
*SsSSVP1* (SS1G_02068)	RNA-silencing	Reduced	Rapeseed	Encoding a cysteine-rich, small protein	Lyu et al. (2016)
*Sscp1* (SS1G_10096)	Knockout	Reduced	*A*.*thaliana*	Encoding a cerato-platanin protein	Yang et al. (2018)
*Ssnsd1* (Sscle16g109570)	Knockout	Reduced	Tomato, celery	GATA-type IVb zinc-finger transcription factor	Li et al. (2018)
*SsSm1* (SS1G_10096)	RNA-silencing	Reduced	Rapeseed, soybean	Encoding a cerato-platanin family protein	Pan et al. (2018)
*Ssams2* (SS1G_03252)	RNA-silencing	Reduced	Soybean	Cell-cycle-regulated GATA transcription factor in eukaryotic organisms	Liu et al. (2018)
*SsSte12* (SS1G_07136)	RNA-silencing	Reduced	Tomato, bush bean	Downstream transcription factor of MAPK pathway	Xu et al. (2018)
*SsC_6_TF1* (Sscle04g036970)	RNA-silencing	Reduced	Pea	C6 transcription factor	Sang et al. (2019)
*SsTrr1* (SS1G_05899)	RNA-silencing	Reduced	*A*.*thaliana*, *N*.*benthamiana*	Thioredoxin reductase	Zhang et al. (2019)
*SsSvf1* (SS1G_01919)	RNA-silencing	Reduced	*A*.*thaliana*, rapeseed	Survival factor1, mediated cell survival under oxidative stress	Yu et al. (2019)
*Sshk*	RNA-silencing	Increased	Rapeseed, cucumber	Histidine kinases	Li et al. (2019)
*SsSaxA* (SS1G_12040)	Knockout	Reduced	*A*.*thaliana*	Isothiocyanate hydrolase	Chen et al. (2020)
*SsATG8* (SsATG8), *SsNBR1* (XP_001594039.1)	Knockout	Reduced	*A*.*thaliana*, tomato	Mediated autophagic degradation	Zhang H. et al., 2021
*SsERP1* (SS1G_11468)	Knockout	Reduced	*N*.*benthamiana*	Ethylene pathway repressor protein 1	Fan et al. (2021b)
*SsCat2* (SS1G_00547)	Knockout	Reduced	*N*.*benthamiana*, rapeseed	Catalase	Huang et al. (2021)
*Ssos4* (SS1G_06598)	Knockout	Reduced	Rapeseed	Encoding a phosphotransferase in the MAPK cascade	Li et al. (2021)
*SsEmp24*, *SsErv25*	Knockout	Reduced	Rapeseed, soybean	Early secretory pathway-related P24 protein	Xie et al. (2021)
*SsYCP1* (SS1G_06230)	overexpression (transient)	Increased	*N.benthamiana*	Yml079-like Cupin protein	Fan et al. (2021a)
*SsTrx1* (SS1G_08534)	RNA-silencing	Reduced	*A*.*thaliana*, *N*.*benthamiana*, rapeseed	Thioredoxin 1, associated with redox regulation and antioxidant defense	Rana et al. (2021)
*Sscnd1* (SS1G_11468)	RNA-silencing	Reduced	Rapeseed	Homologous to MAS protein and participates in the formation of appressorium	Ding et al. (2021)
*Sscle*_*10g079050*	Knockout	Reduced	*A*.*thaliana*, lettuce	Amidase-encoding gene	Li et al. (2022)
*SsNep2* (SS1G_11912)	Knockout	Reduced	*A.thaliana, N.benthamiana*	Encoding necrosis and ethylene-inducing peptides	Yang et al. (2022)
*SsCut1* (SS1G_08104)	Knockout	Reduced	*A.thaliana*, rapeseed	Encoding a cutinase modulated virulence and cutinase activity	Gong et al. (2022)
*SsAtg1* (Sscle_12g087380)	Knockout	Reduced	*N*.*benthamiana*, soybean	Activating autophagy	Jiao et al. (2022a)
*Sspka2* (Sscle14g098350), *SspkaR* (Sscle09g071910)	Knockout	Reduced	*N*.*benthamiana*, soybean, *Vicia faba*	Two components of cAMP signaling	Yu and Rollins (2022)
*SsFoxE3*	Knockout	Reduced	Soybean, tomato, pepper	Forkhead-box family transcription factor	Jiao et al. (2022b)
*SsFkh1* (SS1G_07360), *SsMkk1* (SS1G_00059), *SsBck1* (SS1G_10983), *SsSmk3* (SS1G_05445), *SsPkc1* (SS1G_14026)	RNA-silencing, knockout Knockout Knockout Knockout Knockout	Reduced Reduced Reduced Reduced Reduced	Tomato, cowpea	Forkhead-containing proteins (Fkh1), MAPK signaling pathway components (Mkk1, Bck1, Smk3, Pck1)	Fan et al. (2017) and Cong et al. (2022)
*SsAGM1* (SS1G_01582)	RNA-silencing	Reduced	*A*.*thaliana*, soybean, tomato	N-acetylglucosamine-phosphate mutase	Zhang et al. (2022)
*SsCox17* (sscle_01g006600)	RNA-silencing	Reduced	*A*.*thaliana*, rapeseed	A copper chaperone shuttled copper ions from the cytosol to the mitochondria for the cytochrome c oxidase assembly	Ding et al. (2022)
*SsMrt4* (SS1G_11436)	Knockout	Abolished	*A.thaliana, N.benthamiana*	Ribosome assembly factor	Yang et al. (2023)
*SSA* (sscle_01g001830)	Knockout	Increased	Rapeseed	Encoding agglutinin protein	Wang et al. (2023)
*SsGSR1* (SS1G_11413)	Knockout	Reduced	*N.benthamiana*, rapeseed	Glycosylphosphatidylinositol-anchored protein	Hu et al. (2023)
*SsNR* (SS1G_01885)	RNA-silencing	Reduced	Rapeseed, soybean	Nitrate reductase	Wei et al. (2023)
*SsTOR* (Sscle_02g011660)	RNA-silencing	Reduced	Tomato, cowpea, pepper	Key components of the TOR signaling pathway	Jiao et al. (2023)
*SsCak1* (Sscle_11g085070)	UV-mutagenized	Abolished	*A.thaliana, N.benthamiana*	Protein kinase has a conserved eukaryotic kinase domain	Qin et al. (2023)
*Sspde2* (Sscle06g053640)	UV-mutagenized	Reduced	*A*.*thaliana*, *N*.*benthamiana*	Encoding a cAMP phosphodiesterase	Xu et al. (2023)
*SsGAP1* (sscle_12g 086880), *SsRAS1* (Sscle10g080240), *SsRAS2* (Sscle09g072910)	UV-mutagenized, knockout, knockout	Reduced	*A.thaliana, N.benthamiana*	RAS signalling components	Xu et al. (2024)
*SsSte50* (sscle_07g058440), *SsSte11* (sscle_03g025710), *SsSte7* (sscle_09g069880), *Smk1* (sscle_12g090900), *SsSte12* (sscle_06g051560)	UV-mutagenized, UV-mutagenized, Knockout, Knockout, Knockout	Abolished, Abolished, Abolished, Abolished, Reduced	*A.thaliana, N.benthamiana*	MAPK signaling pathway components	Tian et al. (2024)

The signaling and regulatory factors of *S. sclerotiorum* involve the regulation of multiple genes. *SsTOR* is responsible for controlling cell wall integrity and virulence, and it participates in the rapamycin target signaling pathway (Jiao et al., 2023). *SsCat2* regulates catalase activity, affecting aspects such as cell membrane dryness and pathogenicity (Huang et al., 2021). *SsPKA2* and *SsPKAR* are involved in cAMP signaling, playing roles in growth and virulence (Yu and Rollins, 2022). Moreover, *SsAMS2* contains a GATA-box domain, regulating hyphal growth, adhesion structure formation, and virulence (Liu et al., 2018). Lastly, *SsSm1* participates in the MAPK signaling pathway, influencing aspects such as hyphal growth and pathogenicity (Pan et al., 2018).

These genes play critical roles in the metabolic regulation of *S. sclerotiorum*. *Ssoah1* regulates oxalic acid content, affecting the toxicity of *S. sclerotiorum* (Liang et al., 2015a; Rana et al., 2022). Next, *SsSOD1* is responsible for regulating the clearance capacity of ROS, directly impacting the pathogenicity of *S. sclerotiorum*. Additionally, *SsODC1* and *SsODC2* are involved in oxalic acid metabolism regulation, playing crucial regulatory roles in forming infection cushions and virulence during the infection process (Xu and Chen, 2013).

In the regulation of cellular structure and function in *S. sclerotiorum*, *SsAGM1* participates in chitin synthesis, influencing cell wall structure and sclerotia formation (Zhang et al., 2022). Subsequently, *SsGSR1* encodes glutathione sulfurtransferase, regulating cell wall integrity and the pathogenicity of *S. sclerotiorum* (Hu et al., 2023). *SsCP1* encodes the Cerato-platanin protein, affecting cell death and invasion ability during infection (Yang et al., 2018). Furthermore, as a small secreted protein, SsSSVP1 influences plant cell death and the infection of *S. sclerotiorum* (Lyu et al., 2016).

In the adaptation to environmental changes and stress responses in *S. sclerotiorum*, SsNE2, as a novel necrosis-inducing protein, influences the fungal growth, pathogenicity, and ability to respond to environmental stress (Seifbarghi et al., 2020). As we know, thioredoxin reductases play crucial roles in maintaining cellular redox homeostasis. *SsTrr1*, encoding thioredoxin reductase, participates in oxidative stress responses, thereby affecting the pathogenicity of *S. sclerotiorum* (Zhang et al., 2019). Additionally, SsEmp24 and SsErv25, as early secretory pathway-related proteins, regulate the protein secretion process, playing important roles in the pathogenicity of *S. sclerotiorum*. Their absence leads to abnormal fungal growth, sclerotium formation, formation of appressorium, and lower virulence in host plants (Xie et al., 2021).

## 5 Host-induced gene silencing of virulence-related genes of *Sclerotinia Sclerotiorum*

Most eukaryotic organisms possess the RNA interference (RNAi) pathway. In this process, double-stranded RNA (dsRNA) can be processed into small interfering RNA (siRNA), which can induce gene silencing. As RNA can be transferred from plant hosts to related eukaryotic pathogens, the RNAi pathway has been utilized for HIGS to control pathogens. In this scenario, hosts are engineered to express dsRNAs targeting crucial pathogen genes, thus disrupting their successful lifecycle. It is considered to be a potentially effective tool for controlling various fungi in crops (Chen et al., 2016; Zhu et al., 2017; Qi et al., 2018).

With the discovery of more and more virulence-related genes of *S. sclerotiorum*, many genes can be used to control *S. sclerotiorum* through HIGS. In tobacco, the authors tested whether the MAPK cascade consisting of SsSte50-SsSte11-SsSte7-Smk1 could serve as a target for disease control in HIGS. *SsSte50* is used to make hairpin RNAi constructs. Compared with no-load, the lesion area was significantly reduced. The data showed that the HIGS of *SsSte50* was heritable and could be used as a good target for controlling *S. sclerotiorum* (Tian et al., 2024). Besides, a gene was found to encode a cAMP phosphodiesterase (*SsPDE2*). The author introduced the construction of HIGS targeting *SsPDE2* into *N.benthamiana*, observing a significant reduction in virulence against *S. sclerotiorum*. In summary, *SsPDE2* may serve as a HIGS target to control stem rot in the field (Xu et al., 2023). Transient silencing of *Sscnd1* gene by HIGS mediated by tobacco rattle virus (TRV) can significantly reduce the occurrence of tobacco diseases. Three transgenic *Arabidopsis* lines with HIGS gene showed a high level of resistance to *S. sclerotiorum* and reduced the expression of *Sscnd1* (Ding et al., 2021). Similarly, a putative protein kinase SsCak1, silencing by TRV-HIGS can reduce virulence and enhance host resistance to *S. sclerotiorum* (Qin et al., 2023). In rapeseed, three disease-causing genes, the endo-polygalacturonase gene (*SsPG1*), cellobiohydrolase gene (*SsCBH*), and oxaloacetate acetylhydrolase gene (*SsOAH1*), were selected as the targets of HIGS, which significantly reduced the transcript levels of the target genes (Wu et al., 2022). A RAS-GTPase activating protein SsGAP1, which plays an important role in sclerotia formation, showed complex appressorium production and virulence. The author observed reduced virulence when they introduced HIGS constructs targeting *SsGAP1*, *SsRAS1*, and *SsRAS2* in Arabidopsis, as well as in tobacco (Xu et al., 2024). Likewise, *S. sclerotiorum* thioredoxin 1 gene (*SsTrx1*) has also been identified as a potential HIGS target through the disease resistance analysis in above two plants (Rana et al., 2021).

## 6 Conclusion

The extremely dangerous soil-borne pathogen *S. sclerotiorum* poses a serious risk to crops used in agricultural production. *S. sclerotiorum* can not be eradicated in the field due to the lack of resistant breeding and the resistance of *S. sclerotiorum* strains to fungicides. On the other hand, the negative effects of pesticide residues and environmental pollution are also readily apparent, as they can result in the emergence of drug-resistant strains in the field and the death of beneficial organisms. Thus, it will be helpful to regulate *S. sclerotiorum* to comprehend the pathophysiology of the organism as well as to create and employ disease-resistant genes in plants. For instance, oxalic acid is a pathogenic component of *S. sclerotiorum.* Hence, lowering the pathogenicity of *S. sclerotiorum* can be achieved by reducing oxalic acid production. Moreover, targeting silencing of some proteins, such as Ss-Rhs1, can lead to aberrant colony formation and decreased pathogenicity to the host plant, hence improving host plant resistance to *S. sclerotiorum*. However, for all control methods, a successful strategy should be based on an in-depth understanding of the pathogenesis of *S.sclerotiorum*. The application of HIGS targets of known virulence-related genes can effectively provide host resistance to *S. sclerotiorum.* This may be an effective way to control sclerotinia in the future, providing a new idea for the green control of this broad host range plant pathogen.

## Author contributions

YZ: Writing – original draft. CW: Writing – original draft. YD: Writing – original draft. WY: Writing – original draft. TZ: Writing – original draft. JL: Writing – review & editing, Writing – original draft.

## References

[ref1] Bateman D. F. Beer S. V. (1965). Simultaneous production and synergistic action of oxalic acid and polygalacturonase during pathogenesis by Sclerotium rolfsii. Phytopathology 55, 204–211, PMID: 14274523

[ref2] Behnam S. Ahmadzadeh M. Sharifi Tehrani A. Hedjaroude G. A. Farzaneh M. (2007). Biological control of Sclerotinia sclerotiorum (lib.) de Bary, the causal agent of white mold, by pseudomonas species on canola petals. Commun. Agric. Appl. Biol. Sci. 72, 993–996, PMID: 18396840

[ref3] Boland G. J. Hall R. (1994). Index of plant hosts of Sclerotinia sclerotiorum. Can. J. Plant Pathol. 16, 93–108. doi: 10.1080/07060669409500766

[ref4] Bolton M. D. Thomma B. P. H. J. Nelson B. D. (2006). Sclerotinia sclerotiorum (lib.) de Bary: biology and molecular traits of a cosmopolitan pathogen. Mol. Plant Pathol. 7, 1–16. doi: 10.1111/j.1364-3703.2005.00316.x 20507424

[ref5] Chen W. Kastner C. Nowara D. Oliveira-Garcia E. Rutten T. Zhao Y.. (2016). Host-induced silencing of fusarium culmorum genes protects wheat from infection. J. Exp. Bot. 67, 4979–4991. doi: 10.1093/jxb/erw263, PMID: 27540093 PMC5014151

[ref6] Chen J. Ullah C. Reichelt M. Beran F. Yang Z.-L. Gershenzon J.. (2020). The phytopathogenic fungus Sclerotinia sclerotiorum detoxifies plant glucosinolate hydrolysis products via an isothiocyanate hydrolase. Nat. Commun. 11:3090. doi: 10.1038/s41467-020-16921-2, PMID: 32555161 PMC7303113

[ref7] Clarkson J. P. Phelps K. Whipps J. M. Young C. S. Smith J. A. Watling M. (2004). Forecasting sclerotinia disease on lettuce: toward developing a prediction model for carpogenic germination of sclerotia. Phytopathology 94, 268–279. doi: 10.1094/PHYTO.2004.94.3.268 18943975

[ref8] Cong J. Xiao K. Jiao W. Zhang C. Zhang X. Liu J.. (2022). The coupling between Cell Wall integrity mediated by MAPK kinases and SsFkh1 is involved in Sclerotia formation and pathogenicity of Sclerotinia sclerotiorum. Front. Microbiol. 13:816091. doi: 10.3389/fmicb.2022.816091, PMID: 35547112 PMC9081980

[ref9] Dallal Bashi Z. Hegedus D. D. Buchwaldt L. Rimmer S. R. Borhan M. H. (2010). Expression and regulation of Sclerotinia sclerotiorum necrosis and ethylene-inducing peptides (NEPs). Mol. Plant Pathol. 11, 43–53. doi: 10.1111/j.1364-3703.2009.00571.x 20078775 PMC6640525

[ref10] Dallal Bashi Z. Rimmer S. R. Khachatourians G. G. Hegedus D. D. (2012). Factors governing the regulation of Sclerotinia sclerotiorum cutinase a and polygalacturonase 1 during different stages of infection. Can. J. Microbiol. 58, 605–616. doi: 10.1139/w2012-031, PMID: 22524557

[ref11] Ding Y. Chen Y. Wu Z. Yang N. Rana K. Meng X.. (2022). SsCox17, a copper chaperone, is required for pathogenic process and oxidative stress tolerance of Sclerotinia sclerotiorum. Plant Sci. 322:111345. doi: 10.1016/j.plantsci.2022.111345, PMID: 35691151

[ref12] Ding Y. Chen Y. Yan B. Liao H. Dong M. Meng X.. (2021). Host-induced gene silencing of a multifunction gene Sscnd1 enhances plant resistance against Sclerotinia sclerotiorum. Front. Microbiol. 12:693334. doi: 10.3389/fmicb.2021.693334, PMID: 34690946 PMC8531507

[ref13] Erental A. Harel A. Yarden O. (2007). Type 2A phosphoprotein phosphatase is required for asexual development and pathogenesis of Sclerotinia sclerotiorum. Mol. Plant-Microbe Interact. 20, 944–954. doi: 10.1094/MPMI-20-8-0944, PMID: 17722698

[ref14] Fan H. Yang W. Nie J. Lin C. Wu J. Wu D.. (2021a). Characterization of a secretory YML079-like Cupin protein that contributes to Sclerotinia sclerotiorum pathogenicity. Microorganisms 9:2519. doi: 10.3390/microorganisms9122519, PMID: 34946121 PMC8704077

[ref15] Fan H. Yang W. Nie J. Zhang W. Wu J. Wu D.. (2021b). A novel effector protein SsERP1 inhibits plant ethylene Signaling to promote Sclerotinia sclerotiorum infection. J. Fungi 7:825. doi: 10.3390/jof7100825, PMID: 34682246 PMC8537369

[ref16] Fan H. Yu G. Liu Y. Zhang X. Liu J. Zhang Y.. (2017). An atypical forkhead-containing transcription factor SsFKH1 is involved in sclerotial formation and is essential for pathogenicity in Sclerotinia sclerotiorum. Mol. Plant Pathol. 18, 963–975. doi: 10.1111/mpp.12453, PMID: 27353472 PMC6638265

[ref17] Fernando W. G. D. Nakkeeran S. Zhang Y. Savchuk S. (2007). Biological control of *Sclerotinia sclerotiorum* (lib.) de Bary by *pseudomonas* and *bacillus* species on canola petals. Crop Prot. 26, 100–107. doi: 10.1016/j.cropro.2006.04.007

[ref18] Godoy G. Steadman J. R. Dickman M. B. Dam R. (1990). Use of mutants to demonstrate the role of oxalic acid in pathogenicity of *Sclerotinia sclerotiorum* on *Phaseolus vulgaris*. Physiol. Mol. Plant Pathol. 37, 179–191. doi: 10.1016/0885-5765(90)90010-U

[ref19] Gong Y. Fu Y. Xie J. Li B. Chen T. Lin Y.. (2022). Sclerotinia sclerotiorum SsCut1 modulates virulence and Cutinase activity. J. Fungi 8:526. doi: 10.3390/jof8050526, PMID: 35628781 PMC9143608

[ref20] Guimarães R. L. Stotz H. U. (2004). Oxalate production by Sclerotinia sclerotiorum deregulates guard cells during infection. Plant Physiol. 136, 3703–3711. doi: 10.1104/pp.104.049650, PMID: 15502012 PMC527168

[ref21] Heffer L. V. Johnson K. B. (2007). *White mold (Sclerotinia)*. The Plant Health Instructor. doi: 10.1094/PHI-I-2007-0809-01

[ref22] Hegedus D. D. Rimmer S. R. (2005). Sclerotinia sclerotiorum: when “to be or not to be” a pathogen? FEMS Microbiol. Lett. 251, 177–184. doi: 10.1016/j.femsle.2005.07.040 16112822

[ref23] Heller A. Witt-Geiges T. (2013). Oxalic acid has an additional, detoxifying function in Sclerotinia sclerotiorum pathogenesis. PLoS One 8:e72292. doi: 10.1371/journal.pone.0072292, PMID: 23951305 PMC3741138

[ref24] Hu Y. Gong H. Lu Z. Zhang P. Zheng S. Wang J.. (2023). Variable tandem glycine-rich repeats contribute to cell death-inducing activity of a glycosylphosphatidylinositol-anchored Cell Wall protein that is associated with the pathogenicity of Sclerotinia sclerotiorum. Microbiol. Spectr. 11:e0098623. doi: 10.1128/spectrum.00986-23, PMID: 37140432 PMC10269696

[ref25] Huang Z. Lu J. Liu R. Wang P. Hu Y. Fang A.. (2021). SsCat2 encodes a catalase that is critical for the antioxidant response, QoI fungicide sensitivity, and pathogenicity of Sclerotinia sclerotiorum. Fungal Genet. Biol. 149:103530. doi: 10.1016/j.fgb.2021.103530, PMID: 33561548

[ref26] Jahan R. Siddique S. S. Jannat R. Hossain M. M. (2022). Cosmos white rot: first characterization, physiology, host range, disease resistance, and chemical control. J. Basic Microbiol. 62, 911–929. doi: 10.1002/jobm.202200098 35642304

[ref27] Jiao W. Ding W. Rollins J. A. Liu J. Zhang Y. Zhang X.. (2023). Cross-talk and multiple control of target of rapamycin (TOR) in Sclerotinia sclerotiorum. Microbiol. Spectr. 11:e0001323. doi: 10.1128/spectrum.00013-23, PMID: 36943069 PMC10100786

[ref28] Jiao W. Yu H. Chen X. Xiao K. Jia D. Wang F.. (2022a). The SsAtg1 activating autophagy is required for Sclerotia formation and pathogenicity in Sclerotinia sclerotiorum. J. Fungi (Basel) 8:1314. doi: 10.3390/jof8121314, PMID: 36547647 PMC9787769

[ref29] Jiao W. Yu H. Cong J. Xiao K. Zhang X. Liu J.. (2022b). Transcription factor SsFoxE3 activating SsAtg8 is critical for sclerotia, compound appressoria formation, and pathogenicity in Sclerotinia sclerotiorum. Mol. Plant Pathol. 23, 204–217. doi: 10.1111/mpp.13154, PMID: 34699137 PMC8743022

[ref30] Jurick W. M. Rollins J. A. (2007). Deletion of the adenylate cyclase (sac1) gene affects multiple developmental pathways and pathogenicity in Sclerotinia sclerotiorum. Fungal Genet. Biol. 44, 521–530. doi: 10.1016/j.fgb.2006.11.005, PMID: 17178247

[ref31] Kim Y. T. Prusky D. Rollins J. A. (2007). An activating mutation of the Sclerotinia sclerotiorum pac1 gene increases oxalic acid production at low pH but decreases virulence. Mol. Plant Pathol. 8, 611–622. doi: 10.1111/j.1364-3703.2007.00423.x, PMID: 20507525

[ref32] Lehner M. S. de Paula Júnior T. J. Del Ponte E. M. Mizubuti E. S. G. Pethybridge S. J. (2017). Independently founded populations of Sclerotinia sclerotiorum from a tropical and a temperate region have similar genetic structure. PLoS One 12:e0173915. doi: 10.1371/journal.pone.0173915, PMID: 28296968 PMC5352009

[ref33] Li X. Guo M. Xu D. Chen F. Zhang H. Pan Y.. (2015). The nascent-polypeptide-associated complex alpha subunit regulates the polygalacturonases expression negatively and influences the pathogenicity of Sclerotinia sclerotiorum. Mycologia 107, 1130–1137. doi: 10.3852/14-250, PMID: 26297780

[ref34] Li M. Liang X. Rollins J. A. (2012). Sclerotinia sclerotiorum γ-glutamyl transpeptidase (Ss-Ggt1) is required for regulating glutathione accumulation and development of sclerotia and compound appressoria. Mol. Plant-Microbe Interact. 25, 412–420. doi: 10.1094/MPMI-06-11-0159 22046959

[ref35] Li W. Lu J. Yang C. Arildsen K. Li X. Xia S. (2022). An amidase contributes to full virulence of Sclerotinia sclerotiorum. Int. J. Mol. Sci. 23:11207. doi: 10.3390/ijms231911207, PMID: 36232508 PMC9570306

[ref36] Li J. Mu W. Veluchamy S. Liu Y. Zhang Y. Pan H.. (2018). The GATA-type IVb zinc-finger transcription factor SsNsd1 regulates asexual-sexual development and appressoria formation in Sclerotinia sclerotiorum. Mol. Plant Pathol. 19, 1679–1689. doi: 10.1111/mpp.12651, PMID: 29227022 PMC6638148

[ref37] Li R. Rimmer R. Buchwaldt L. Sharpe A. G. Séguin-Swartz G. Hegedus D. D. (2004). Interaction of Sclerotinia sclerotiorum with *Brassica napus*: cloning and characterization of endo- and exo-polygalacturonases expressed during saprophytic and parasitic modes. Fungal Genet. Biol. 41, 754–765. doi: 10.1016/j.fgb.2004.03.002, PMID: 15219560

[ref38] Li T. Xiu Q. Wang J. Duan Y. Zhou M. (2021). A putative MAPK kinase kinase gene Ssos4 is involved in mycelial growth, virulence, osmotic adaptation, and sensitivity to Fludioxonil and is essential for SsHog1 phosphorylation in Sclerotinia sclerotiorum. Phytopathology 111, 521–530. doi: 10.1094/PHYTO-07-20-0292-R, PMID: 33044134

[ref39] Li J. Zhu F. Li J. (2019). Expression of the histidine kinase gene Sshk correlates with Dimethachlone resistance in Sclerotinia sclerotiorum. Phytopathology 109, 395–401. doi: 10.1094/PHYTO-05-18-0156-R 30070619

[ref40] Liang X. Liberti D. Li M. Kim Y.-T. Hutchens A. Wilson R.. (2015a). Oxaloacetate acetylhydrolase gene mutants of Sclerotinia sclerotiorum do not accumulate oxalic acid, but do produce limited lesions on host plants. Mol. Plant Pathol. 16, 559–571. doi: 10.1111/mpp.12211, PMID: 25285668 PMC6638444

[ref41] Liang X. Moomaw E. W. Rollins J. A. (2015b). Fungal oxalate decarboxylase activity contributes to Sclerotinia sclerotiorum early infection by affecting both compound appressoria development and function. Mol. Plant Pathol. 16, 825–836. doi: 10.1111/mpp.12239, PMID: 25597873 PMC6638544

[ref42] Liberti D. Rollins J. A. Dobinson K. F. (2013). Peroxysomal carnitine acetyl transferase influences host colonization capacity in Sclerotinia sclerotiorum. Mol. Plant-Microbe Interact. 26, 768–780. doi: 10.1094/MPMI-03-13-0075-R 23581822

[ref43] Liu L. Wang Q. Zhang X. Liu J. Zhang Y. Pan H. (2018). Ssams2, a gene encoding GATA transcription factor, is required for Appressoria formation and chromosome segregation in Sclerotinia sclerotiorum. Front. Microbiol. 9:3031. doi: 10.3389/fmicb.2018.03031, PMID: 30574138 PMC6291475

[ref44] Lyu X. Shen C. Fu Y. Xie J. Jiang D. Li G.. (2015a). Comparative genomic and transcriptional analyses of the carbohydrate-active enzymes and secretomes of phytopathogenic fungi reveal their significant roles during infection and development. Sci. Rep. 5:15565. doi: 10.1038/srep15565, PMID: 26531059 PMC4632110

[ref45] Lyu X. Shen C. Fu Y. Xie J. Jiang D. Li G.. (2015b). The microbial opsin homolog Sop1 is involved in Sclerotinia sclerotiorum development and environmental stress response. Front. Microbiol. 6:1504. doi: 10.3389/fmicb.2015.01504, PMID: 26779159 PMC4703900

[ref46] Lyu X. Shen C. Fu Y. Xie J. Jiang D. Li G.. (2016). A small secreted virulence-related protein is essential for the necrotrophic interactions of Sclerotinia sclerotiorum with its host plants. PLoS Pathog. 12:e1005435. doi: 10.1371/journal.ppat.1005435, PMID: 26828434 PMC4735494

[ref48] Pan Y. Wei J. Yao C. Reng H. Gao Z. (2018). SsSm1, a Cerato-platanin family protein, is involved in the hyphal development and pathogenic process of Sclerotinia sclerotiorum. Plant Sci. 270, 37–46. doi: 10.1016/j.plantsci.2018.02.001, PMID: 29576085

[ref49] Purdy L. H. (1979). Sclerotinia sclerotiorum: history, diseases and symptomatology, host range, geographic distribution, and impact. Phytopathology 69, 875–880. doi: 10.1094/Phyto-69-875

[ref50] Qi T. Zhu X. Tan C. Liu P. Guo J. Kang Z.. (2018). Host-induced gene silencing of an important pathogenicity factor PsCPK1 in Puccinia striiformis f. sp. tritici enhances resistance of wheat to stripe rust. Plant Biotechnol. J. 16, 797–807. doi: 10.1111/pbi.12829, PMID: 28881438 PMC5814584

[ref51] Qin L. Nong J. Cui K. Tang X. Gong X. Xia Y.. (2023). SsCak1 regulates growth and pathogenicity in Sclerotinia sclerotiorum. Int. J. Mol. Sci. 24:12610. doi: 10.3390/ijms241612610, PMID: 37628791 PMC10454577

[ref52] Qu X. Yu B. Liu J. Zhang X. Li G. Zhang D.. (2014). MADS-box transcription factor SsMADS is involved in regulating growth and virulence in Sclerotinia sclerotiorum. Int. J. Mol. Sci. 15, 8049–8062. doi: 10.3390/ijms15058049, PMID: 24815067 PMC4057718

[ref53] Rana K. Ding Y. Banga S. S. Liao H. Zhao S. Yu Y.. (2021). Sclerotinia sclerotiorum Thioredoxin1 (SsTrx1) is required for pathogenicity and oxidative stress tolerance. Mol. Plant Pathol. 22, 1413–1426. doi: 10.1111/mpp.13127, PMID: 34459563 PMC8518572

[ref54] Rana K. Yuan J. Liao H. Banga S. S. Kumar R. Qian W.. (2022). Host-induced gene silencing reveals the role of Sclerotinia sclerotiorum oxaloacetate acetylhydrolase gene in fungal oxalic acid accumulation and virulence. Microbiol. Res. 258:126981. doi: 10.1016/j.micres.2022.126981 35183041

[ref55] Rollins J. A. (2003). The Sclerotinia sclerotiorum pac1 gene is required for sclerotial development and virulence. Mol. Plant-Microbe Interact. 16, 785–795. doi: 10.1094/MPMI.2003.16.9.785, PMID: 12971602

[ref56] Sang H. Chang H.-X. Chilvers M. I. (2019). A Sclerotinia sclerotiorum transcription factor involved in Sclerotial development and virulence on pea. mSphere 4, e00615–e00618. doi: 10.1128/mSphere.00615-18 30674647 PMC6344603

[ref57] Seifbarghi S. Borhan M. H. Wei Y. Ma L. Coutu C. Bekkaoui D.. (2020). Receptor-like kinases BAK1 and SOBIR1 are required for necrotizing activity of a novel Group of Sclerotinia sclerotiorum necrosis-inducing effectors. Front. Plant Sci. 11:1021. doi: 10.3389/fpls.2020.01021, PMID: 32754179 PMC7367142

[ref58] Sutton D. C. Deverall B. J. (1983). Studies on infection of bean (*Phaseolus vulgaris*) and soybean (*Glycine max*) by ascospores of Sclerotinia sclerotiarum. Plant Pathol. 32, 251–261. doi: 10.1111/j.1365-3059.1983.tb02832.x

[ref59] Tian L. Li J. Xu Y. Qiu Y. Zhang Y. Li X. (2024). A MAP kinase cascade broadly regulates the lifestyle of Sclerotinia sclerotiorum and can be targeted by HIGS for disease control. Plant J. 118, 324–344. doi: 10.1111/tpj.16606, PMID: 38149487

[ref60] Tian B. Xie J. Fu Y. Cheng J. Li B. Chen T.. (2020). A cosmopolitan fungal pathogen of dicots adopts an endophytic lifestyle on cereal crops and protects them from major fungal diseases. ISME J. 14, 3120–3135. doi: 10.1038/s41396-020-00744-6, PMID: 32814863 PMC7784893

[ref61] Wang X. Li Q. Niu X. Chen H. Xu L. Qi C. (2009). Characterization of a canola C2 domain gene that interacts with PG, an effector of the necrotrophic fungus Sclerotinia sclerotiorum. J. Exp. Bot. 60, 2613–2620. doi: 10.1093/jxb/erp104, PMID: 19407339 PMC2692008

[ref62] Wang Y. Xu Y. Wei J. Zhang J. Wu M. Li G.. (2023). Sclerotinia sclerotiorum agglutinin modulates Sclerotial development, pathogenicity and response to abiotic and biotic stresses in different manners. J. Fungi (Basel) 9:737. doi: 10.3390/jof9070737, PMID: 37504726 PMC10381867

[ref63] Wei J. Yao C. Zhu Z. Gao Z. Yang G. Pan Y. (2023). Nitrate reductase is required for sclerotial development and virulence of Sclerotinia sclerotiorum. Front. Plant Sci. 14:1096831. doi: 10.3389/fpls.2023.1096831, PMID: 37342142 PMC10277653

[ref64] Williams J. R. Stelfox D. (1980). Influence of farming practices in Alberta on germination and apothecium production of sclerotia of Sclerotinia sclerotiorum. Can. J. Plant Pathol. 2, 169–172. doi: 10.1080/07060668009501435

[ref65] Wu J. Yin S. Lin L. Liu D. Ren S. Zhang W.. (2022). Host-induced gene silencing of multiple pathogenic factors of Sclerotinia sclerotiorum confers resistance to Sclerotinia rot in *Brassica napus*. Crop J. 10, 661–671. doi: 10.1016/j.cj.2021.08.007

[ref66] Xiao X. Xie J. Cheng J. Li G. Yi X. Jiang D.. (2014). Novel secretory protein Ss-Caf1 of the plant-pathogenic fungus Sclerotinia sclerotiorum is required for host penetration and normal sclerotial development. Mol. Plant-Microbe Interact. 27, 40–55. doi: 10.1094/MPMI-05-13-0145-R, PMID: 24299212

[ref67] Xie C. Shang Q. Mo C. Xiao Y. Wang G. Xie J.. (2021). Early secretory pathway-associated proteins SsEmp24 and SsErv25 are involved in morphogenesis and pathogenicity in a filamentous Phytopathogenic fungus. MBio 12:e0317321. doi: 10.1128/mBio.03173-21 34933451 PMC8689567

[ref68] Xu L. Chen W. (2013). Random T-DNA mutagenesis identifies a cu/Zn superoxide dismutase gene as a virulence factor of Sclerotinia sclerotiorum. Mol. Plant-Microbe Interact. 26, 431–441. doi: 10.1094/MPMI-07-12-0177-R 23252459

[ref69] Xu T. Li J. Yu B. Liu L. Zhang X. Liu J.. (2018). Transcription factor SsSte12 was involved in mycelium growth and development in Sclerotinia sclerotiorum. Front. Microbiol. 9:2476. doi: 10.3389/fmicb.2018.02476, PMID: 30386319 PMC6200020

[ref70] Xu Y. Qiu Y. Zhang Y. Li X. (2023). A cAMP phosphodiesterase is essential for sclerotia formation and virulence in Sclerotinia sclerotiorum. Front. Plant Sci. 14:1175552. doi: 10.3389/fpls.2023.1175552, PMID: 37324679 PMC10264682

[ref71] Xu Y. Tan J. Lu J. Zhang Y. Li X. (2024). RAS signalling genes can be used as host-induced gene silencing targets to control fungal diseases caused by Sclerotinia sclerotiorum and Botrytis cinerea. Plant Biotechnol. J. 22, 262–277. doi: 10.1111/pbi.14184, PMID: 37845842 PMC10754012

[ref72] Yajima W. Liang Y. Kav N. N. V. (2009). Gene disruption of an arabinofuranosidase/beta-xylosidase precursor decreases Sclerotinia sclerotiorum virulence on canola tissue. Mol. Plant-Microbe Interact. 22, 783–789. doi: 10.1094/MPMI-22-7-0783, PMID: 19522560

[ref73] Yang C. Li W. Huang X. Tang X. Qin L. Liu Y.. (2022). SsNEP2 contributes to the virulence of Sclerotinia sclerotiorum. Pathogens 11:446. doi: 10.3390/pathogens11040446, PMID: 35456121 PMC9026538

[ref74] Yang G. Tang L. Gong Y. Xie J. Fu Y. Jiang D.. (2018). A cerato-platanin protein SsCP1 targets plant PR1 and contributes to virulence of Sclerotinia sclerotiorum. New Phytol. 217, 739–755. doi: 10.1111/nph.14842, PMID: 29076546

[ref75] Yang C. Tang L. Qin L. Zhong W. Tang X. Gong X.. (2023). mRNA turnover protein 4 is vital for fungal pathogenicity and response to oxidative stress in Sclerotinia sclerotiorum. Pathogens 12:281. doi: 10.3390/pathogens12020281, PMID: 36839553 PMC9960052

[ref76] Yu Y. Du J. Wang Y. Zhang M. Huang Z. Cai J.. (2019). Survival factor 1 contributes to the oxidative stress response and is required for full virulence of Sclerotinia sclerotiorum. Mol. Plant Pathol. 20, 895–906. doi: 10.1111/mpp.12801, PMID: 31074170 PMC6589728

[ref77] Yu P.-L. Rollins J. A. (2022). The cAMP-dependent protein kinase a pathway perturbs autophagy and plays important roles in development and virulence of Sclerotinia sclerotiorum. Fungal Biol. 126, 20–34. doi: 10.1016/j.funbio.2021.09.004, PMID: 34930556

[ref78] Yu Y. Xiao J. Du J. Yang Y. Bi C. Qing L. (2016). Disruption of the gene encoding Endo-β-1, 4-xylanase affects the growth and virulence of Sclerotinia sclerotiorum. Front. Microbiol. 7:1787. doi: 10.3389/fmicb.2016.01787, PMID: 27891117 PMC5103160

[ref79] Zhang H. Li Y. Lai W. Huang K. Li Y. Wang Z.. (2021). SsATG8 and SsNBR1 mediated-autophagy is required for fungal development, proteasomal stress response and virulence in Sclerotinia sclerotiorum. Fungal Genet. Biol. 157:103632. doi: 10.1016/j.fgb.2021.103632, PMID: 34710583

[ref80] Zhang M. Liu Y. Li Z. She Z. Chai M. Aslam M.. (2021). The bZIP transcription factor GmbZIP15 facilitates resistance against Sclerotinia sclerotiorum and Phytophthora sojae infection in soybean. iScience 24:102642. doi: 10.1016/j.isci.2021.102642, PMID: 34151234 PMC8188564

[ref81] Zhang J. Wang Y. Du J. Huang Z. Fang A. Yang Y.. (2019). Sclerotinia sclerotiorum Thioredoxin reductase is required for oxidative stress tolerance, virulence, and Sclerotial development. Front. Microbiol. 10:233. doi: 10.3389/fmicb.2019.00233, PMID: 30837967 PMC6382746

[ref82] Zhang J. Xiao K. Li M. Hu H. Zhang X. Liu J.. (2022). SsAGM1-mediated uridine diphosphate-N-Acetylglucosamine synthesis is essential for development, stress response, and pathogenicity of Sclerotinia sclerotiorum. Front. Microbiol. 13:938784. doi: 10.3389/fmicb.2022.938784 35814696 PMC9260252

[ref83] Zhu X. Qi T. Yang Q. He F. Tan C. Ma W.. (2017). Host-induced gene silencing of the MAPKK gene PsFUZ7 confers stable resistance to wheat stripe rust. Plant Physiol. 175, 1853–1863. doi: 10.1104/pp.17.01223, PMID: 29070517 PMC5717739

